# The Fire Resistance Performance of Recycled Aggregate Concrete Columns with Different Concrete Compressive Strengths

**DOI:** 10.3390/ma7127843

**Published:** 2014-12-08

**Authors:** Hongying Dong, Wanlin Cao, Jianhui Bian, Jianwei Zhang

**Affiliations:** 1 College of Architecture and Civil Engineering, Beijing University of Technology, Beijing 100124, China; E-Mails: donghy@bjut.edu.cn (H.D.); zhangjw@bjut.edu.cn (J.Z.); 2 Tianjin Cement Industry Design & Research Institute Co., Ltd., Tianjin 300400, China; E-Mail: bianjianhui1985@126.com

**Keywords:** recycled aggregate concrete (RAC) column, fire resistance, high-temperature test, temperature field, finite element method (FEM) analysis, concrete compressive strength

## Abstract

In order to ascertain the fire resistance performance of recycled aggregate concrete (RAC) components with different concrete compressive strengths, four full-scaled concrete columns were designed and tested under high temperature. Two of the four specimens were constructed by normal concrete with compressive strength ratings of C20 and C30, respectively, while the others were made from recycled coarse aggregate (RCA) concrete of C30 and C40, respectively. Identical constant axial forces were applied to specimens while being subjected to simulated building fire conditions in a laboratory furnace. Several parameters from the experimental results were comparatively analyzed, including the temperature change, vertical displacement, lateral deflection, fire endurance, and failure characteristics of specimens. The temperature field of specimens was simulated with ABAQUS Software (ABAQUS Inc., Provindence, RI, USA) and the results agreed quite well with those from the experiments. Results show that the rate of heat transfer from the surface to the interior of the column increases with the increase of the concrete’s compressive strength for both RAC columns and normal concrete columns. Under the same initial axial force ratio, for columns with the same cross section, those with lower concrete compressive strengths demonstrate better fire resistance performance. The fire resistance performance of RAC columns is better than that of normal concrete columns, with the same concrete compressive strength.

## 1. Introduction

The loss of human life and property is a significant feature of in-building fire disasters. Statistics show that the most common and serious losses in connection with fires occur during building structure fires. Reinforced concrete (RC) construction accounts for a large proportion of buildings; during a building fire its structural performance is degraded, structural deformation increases, and the load-carrying capacity declines. Lots of studies focusing on the fire performance of concrete components were conducted. In the 1990s, Lie* et al.* [1,2] and other Canadian scholars carried out fire resistance tests mainly on normal RC columns and concrete-filled steel tubular columns with different cross sections, and published their fire endurance formulae, which are now widely used in the international construction industry. Kodur* et al.* [3,4,5] and Iguchi* et al.* [6] investigated the fire resistance of RC beams and columns made of normal concrete. Gernay* et al.* [7] studied the structural behavior of concrete columns under natural fires.

Recycled aggregate concrete (RAC), a new type of environmentally friendly material using waste concrete as recycled aggregates to substitute for natural aggregates, is made partly or totally by processes of washing, crushing, grading, and proportional mixing. Although RAC is no longer considered a new technology and many studies have focused on its use, currently most of the experimental studies on RAC fire resistance performance have been confined to RAC material rather than structural components [8,9,10,11,12,13,14]. Xiao* et al.* [15] analyzed the factors affecting thermal conductivity of RAC and provided the calculation method and formula for RAC thermal performance parameters with different amounts of recycled coarse aggregate (RCA) replacement. Vieira* et al.* [16] conducted an experimental study on the residual mechanical performance of concrete produced with RCA after being subjected to high temperatures. Results obtained show that there are no significant differences in the thermal response and post-fire mechanical behavior of concrete made with RCA, when compared to normal concrete. Sarhat* et al.* [17] studied the residual mechanical response of RAC after exposure to elevated temperatures. However, few experimental studies on the fire resistance performance of RAC structures and components have been conducted except when Liu* et al.* [18] investigated the fire resistance performance of the recycled concrete-filled steel tube. Vertical columns are the primary load-carrying structural components in most buildings, and the loss of their load-carrying capacity in a fire may lead to partial or total destruction of the entire structure. Therefore, research into the fire resistance performance of RAC columns is crucial to the preservation of life and property in fire emergencies. In China, Dong* et al.* [19] designed and tested four concrete tubular structure specimens, with one made from normal concrete C20, one made from RCA concrete C20, and the other two made from recycled coarse and fine aggregate concrete, C20 and C40, respectively. Results show that the temperature of specimens with recycled concrete is lower than that of normal concrete in the same position, force conditions, and fire conditions. The load-carrying capacity of specimens with recycled concrete is lower than that of the normal concrete specimens. Fire endurance decreases with the increase of recycled aggregate replacement rate in the specimens.

Considering most columns used in low-rise buildings are concrete columns without an outer steel tube, it is essential to study their fire resistance performance. For this paper, in order to ascertain the fire resistance performance of RAC-constructed components with different concrete compressive strengths, the fire resistance performance of two RAC columns and two normal RC columns with different concrete compressive strengths was analyzed based on experimental data, with the aim of providing an experimental basis for fire resistance design using RAC columns.

## 2. Experimental Details

### 2.1. Model Design

**Figure 1:**
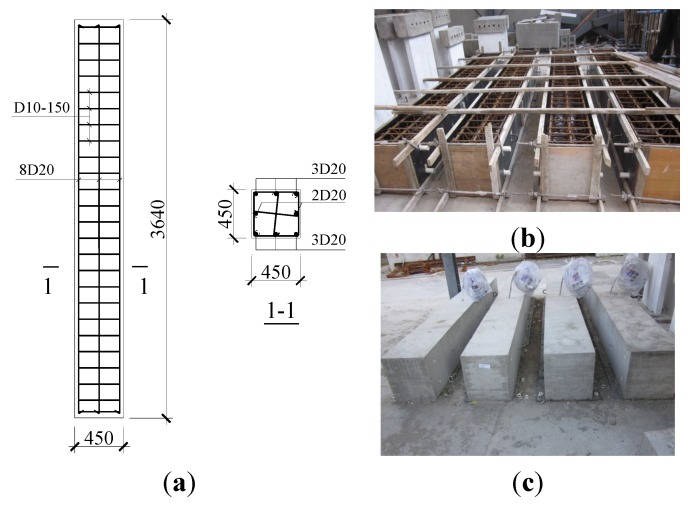
(**a**) Reinforcement details and cross section dimension of columns (mm); (**b**) columns in construction; and (**c**) columns under curing.

Four full-scale concrete columns were constructed: NCC-1, a normal RC column constructed from C20-specified concrete; NCC-2, a normal RC column from C30-specified concrete; RCC-1, a 100% RCA replacement RAC column with C30-specified concrete; and RCC-2, a 100% RCA replacement RAC column using C40-specified concrete. All specimens were constructed outside in winter. The geometry and reinforcement details of the four columns were identical, each with a cross section of 450 mm × 450 mm, a height of 3640 mm, longitudinal bars of 8D20, a ratio of reinforcement of 1.24%, D10-150 stirrups, and a volume stirrup ratio of 0.6%, as shown in Figure 1a. The thickness of the concrete cover was 30 mm for all four columns. The picture of columns in construction and under curing is shown in Figure 1b,c, respectively.

### 2.2. Aggregates, Concrete Mix Design, and Concrete Mechanical Properties

These fine natural aggregates (FA) and coarse natural aggregates (NA) are typical in ready-mix concrete construction in North China. The RCA used in the research came from a local construction and demolition recycling plant. It was made from concrete columns and beams of an old building demolished at Guangqumen, Beijing, China; the test cubic compressive strength was 32.43 MPa. Since the RCA came from demolition, components such as bricks were inevitable (about 1.62 wt%). The RCA used in the research had an aggregate content of 40.1%, mortar aggregate content of 34.9%, residual mortar content of 23.42%, and deleterious material content of 1.62%. The density of the RCA was 2290 kg/m^3^. Its water absorption was 3.23%.

The direct mass replacement method was used to replace NA with RCA. Table 1 shows the dry-weight proportions of the four concrete mix designs in this paper. The RAC was mixed at the site and ready-mixed concrete was used for the normal concrete. A high range water reducer (HRWR) was also used in each normal concrete mix.

**Table 1:** Dry-weight proportions of normal and recycled coarse aggregate (RCA) concrete mixes. FA: fine natural aggregates; NA: coarse natural aggregates; and HRWR: high range water reducer.

Grade level	RCA replacement ratio (%)	Water-to-cement ratio	Water (kg·m^−3^)	Cement (kg·m^−3^)	FA (kg·m^−3^)	NA (kg·m^−3^)	RCA (kg·m^−3^)	HRWR (kg·m^−3^)
C20	0	0.56	107	152	906	1037	-	6.08
C30	0	0.45	111	208	894	990	-	7.56
C30	100	0.56	186	335	576	-	1116	-
C40	100	0.37	178	482	464	-	1124	-

Each grade of concrete after 28 d was tested to ascertain its mechanical properties prior to heating—these results are shown in Table 2. The yielding strength, ultimate strength, and elastic modulus of bar D20 used are 375 MPa, 559 MPa, and 2.02 × 10^5^ MPa, respectively, and those of bar D10 are 433 MPa, 497 MPa, and 1.99 × 10^5^ MPa, respectively.

**Table 2:** Concrete mechanical properties as tested.

Specimen	RCA replacement ratio (%)	Designed concrete level	Test cubic compressive strength (MPa)	Elastic modulus (MPa)
NCC-1	0	C20	17.84	2.34 × 10^4^
NCC-2	0	C30	30.85	3.03 × 10^4^
RCC-1	100	C30	28.57	2.65 × 10^4^
RCC-2	100	C40	40.70	3.15 × 10^4^

### 2.3. Vertical Loading

The vertical load on a building during a fire usually remains constant most of the time. As the vertical load is the main concern in the design of a column, the same axial force ratio was used during the experiments to compare the fire resistance performance of columns with different concrete strengths. The vertical load on the columns was applied using a vertical loading device, which was set under the column and kept static during the fire test. The fire resistance test equipment used limits test duration to a maximum of 4 h, so in order to obtain a complete range of data during the heating process, a high axial force ratio was adopted to accelerate the process of column damage. The axial force ratio, *n*, can be calculated by the following equation:

(1) $$n=\frac{N}{f_{\text{c}}A_{\text{c}}}$$

where *N* is the vertical axial load, which means the vertical load on the two ends of a column herein; *A*_c_ is the cross sectional area of a column and is assumed to remain unchanged unless concrete separates from the column during heating; and *f*_c_ is the design value for concrete axial compressive strength, which is obtained by multiplying the experimental concrete cubic compressive strength by 0.478 according to GB50010-2010 [20] and will decrease as the temperature increases. The experimental vertical load imposed on the columns and the corresponding initial axial force ratios are listed in Table 3. The real axial force ratio, in which the test concrete axial compressive strength is used for *f*_c_, in Equation (1), is also listed in Table 3. The axial force ratios of the four columns are relatively close, as shown in Table 3.

**Table 3:** The vertical load imposed on columns and the corresponding initial axial force ratio.

Specimen	Cross section (mm)	Vertical axial load (kN)	Axial force ratio	Real axial force ratio
NCC-1	450 × 450	3000	1.737	0.830
NCC-2		5250	1.758	0.840
RCC-1		4800	1.736	0.830
RCC-2		6900	1.751	0.837

### 2.4. Loading Equipment and the Heating Curves

**Figure 2:**
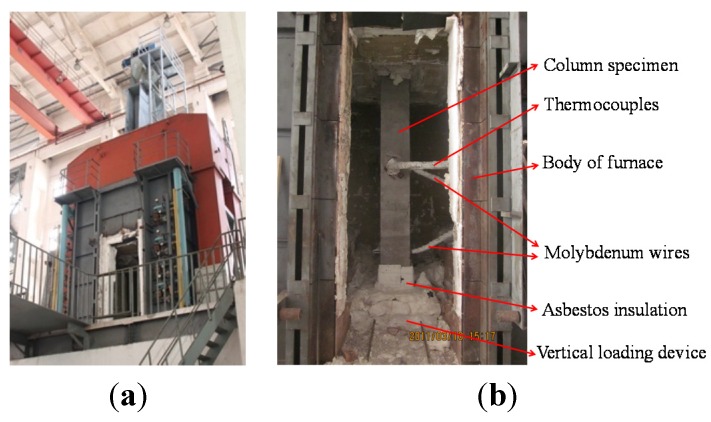
(**a**) Test furnace; and (**b**) test set-up.

The test was carried out in the test column furnace at the Testing Center of the National Fixed Fire Extinguishing System and Fire-Resistant Component Quality Supervision, Tianjin Fire Research Institute, China Ministry of Public Security (Tianjin, China). The test furnace is shown in Figure 2a. There are 13 orifices on the body of the furnace for attaching thermocouplings so as to obtain the temperature inside in real time. To ensure uniformity of temperature within the furnace during heating, the diesel nozzles in the combustion furnace were arranged spirally in order to generate a spiral-shaped heat source. Both ends of the column were hinged to the flanges inside the furnace. The distance between the unaffected parts, which were protected by asbestos insulation, and the fire at each end was 320 mm. Thus, the actual height of the middle part that was in the fire of 3000 mm. The test set-up is shown in Figure 2b.

**Figure 3:**
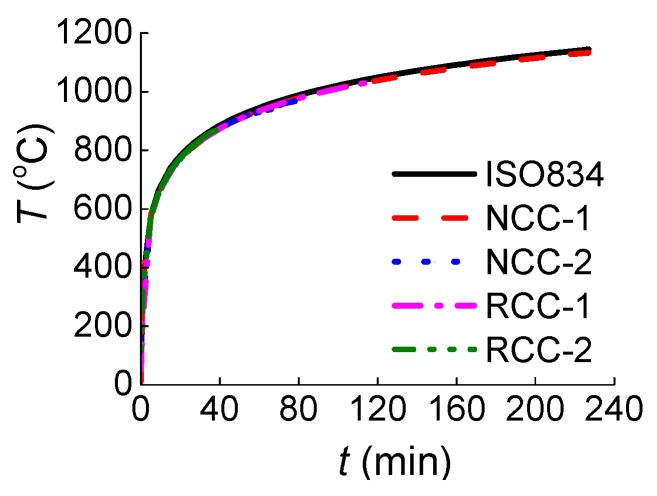
Comparison curves between the actual temperature and ISO834 heating curve.

According to International Standard ISO834 [21], the heating curve formula *T* = *T*_0_ + 345lg(8*t* + 1) was used to control the heating. Here, *T* is the heating temperature in Celsius, *T*_0_ is the initial temperature in Celsius of the furnace, and *t* stands for the heating time in minutes. The actual heating curve, as shown in Figure 3, agrees very well with the one in ISO834 [21].

**Figure 4:**
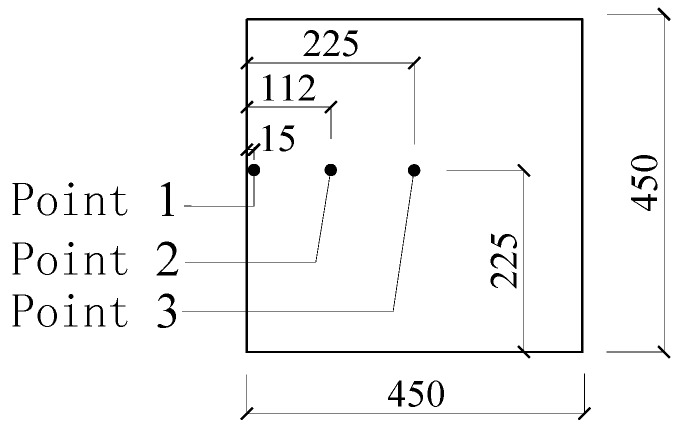
Thermocouple layout diagram (mm).

The specimens were allowed to cool down naturally after the heating test. The thermocouples used were of nickel-chromium or nickel-silicon, and chromel construction; three of these were arranged in the middle cross section along the height of the column, with a distance from the outer side of 15 mm, 112 mm, and 225 mm, respectively, as shown in Figure 4. The main test method and content are: (1) the end of the fire resistance duration was indicated by a sudden drop in vertical load; (2) the temperature inside the cross section was measured by the thermocouples; (3) the axial deformation of the column was obtained using a vertical displacement meter, which was set under the bottom of the column; (4) the lateral deflection at the height of 320 mm and 1820 mm from the bottom of the column was obtained using two molybdenum wires, which were connected to the column through orifices in the furnace; and (5) the failure process was also observed through the observation port on the furnace. There was only one fixed port and only the lower part of the column in furnace could be seen.

## 3. Results and Discussion

### 3.1. Test Results and Analysis

#### 3.1.1. Fire Resistance Duration

The initial vertical load was determined by a fixed axial force ratio and remained unchanged during the heating test. The heating curve from ISO834 was adopted inside the furnace. According to Chinese Standard GB/T 9978-2008 [22], the fire resistance duration of the specimen, meaning the time from the start of the test until the specimen fails, is determined when the axial deformation becomes greater than or equal to *H*/100 (mm), or when the rate of the axial deformation reaches 3*H*/1000 (mm/min). *H* is the valid height of a column on fire, which is 3000 mm here. In this instance, the fire resistance duration of a column was determined to be when the axial deformation is greater than or equal to 30 mm, or when the rate of the axial deformation reaches 9 mm/min.

**Figure 5:**
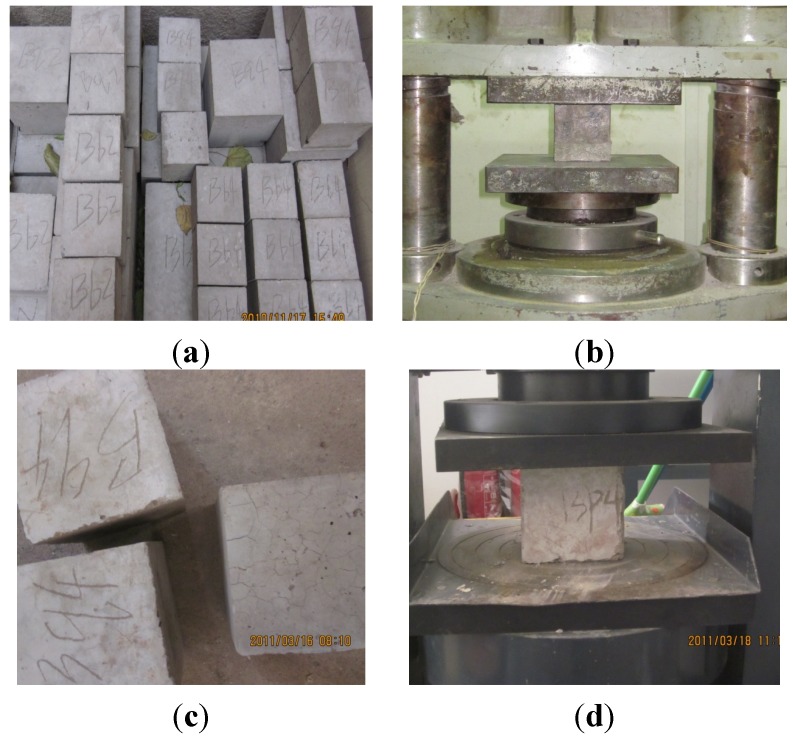
The cubic compressive strength test before and after fire: (**a**) the cubic concrete specimens before fire; (**b**) testing before fire; (**c**) the cubic concrete specimens after fire; and (**d**) testing after fire.

The experimental fire resistance duration of specimens NCC-1, NCC-2, RCC-1, and RCC-2 is 227 min, 78 min, 118 min, and 39 min, respectively. All of the specimens failed because of the large axial deformation. The concrete cubic specimens were put into the furnace with the columns at the same time and subjected to International Organization for Standardization (ISO) fire exposure. The cubic compressive strength after fire was also tested; Figure 5 shows the cubic compressive strength test before and after fire.

Table 4 listed the residual cubic compressive strength and its loss rate *n*_loss_ after fire tests.

**Table 4:** The cubic compressive strength and its loss rate after fire tests.

Specimen	Fire resistance duration (min)	Cubic compressive strength before fire test (MPa)	Residual cubic compressive strength after fire test (MPa)	Loss rate of cubic compressive strength after fire test (%)
NCC-1	227	17.84	0	100.0
NCC-2	78	30.85	16.14	47.7
RCC-1	118	28.57	10.06	64.8
RCC-2	39	40.70	35.20	13.5

**Figure 6:**
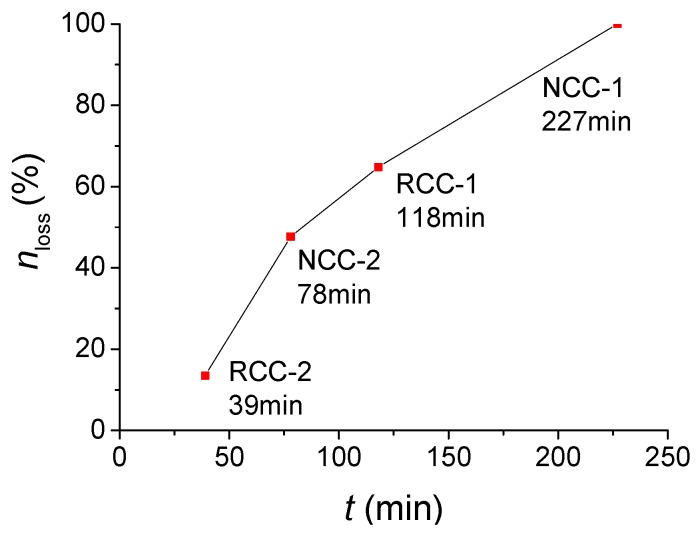
The relationship of loss rate of cubic compressive strength to time.

Figure 6 shows the relationship of loss rate of cubic compressive strength to time. The loss rate of cubic compressive strength after fire tests of both normal concrete and RCA concrete increased with the increasing of the fire time. Concrete is a kind of thermal inertia material [23]. At the early heating time, the temperature inside the concrete was not high enough to make the inner hydrate decompose. The loss of strength was mainly because of the cracks induced by the temperature differences between the outside and inside of columns. With an increase in heating time, the temperature differences between the outside and inside of columns decreased. The hydrate, which worked as the bones of the concrete, started to decompose. This made the concrete strength drop fast. For NCC-1, after 227 min under fire, almost all of the hydrate decomposed and the concrete strength came mainly from the friction force between coarse aggregates and mortar. The concrete cubic specimens of NCC-1 totally lost their strength and their cubic compressive strength dropped to 0. With the reinforcements inside, the column NCC-1 could still stand when it failed.

#### 3.1.2. Temperature at the Measured Points

**Figure 7:**
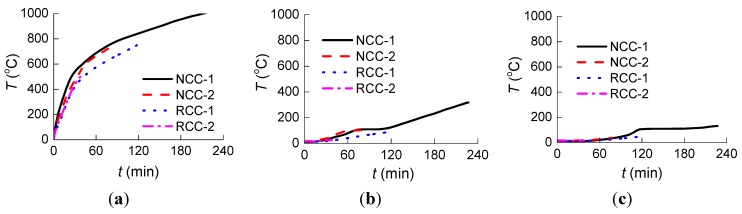
Measured temperature* vs.* time for four specimens at the three measure points: (**a**) Measure Point 1; (**b**) Measure Point 2; and (**c**) Measure Point 3.

The initial temperature inside the furnace was 20 °C. Figure 7 shows the curves of “measured temperature to time” at the three measure points for each of the four specimens.

**Figure 8:**
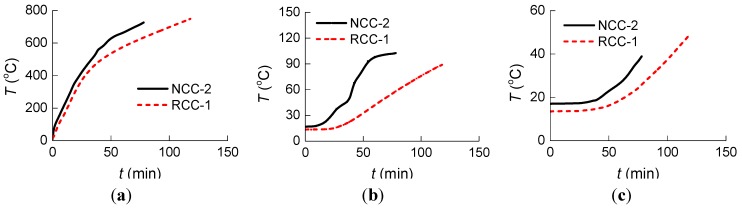
Measured temperature* vs.* time for NCC-2 and RCC-1 at the three measure points: (**a**) Measure Point 1; (**b**) Measure Point 2; and (**c**) Measure Point 3.

Figure 8 shows the curves of “measured temperature to time” at the three measure points of specimen NCC-2 and RCC-1.

In order to compare the temperature at a given time in the columns as a function of the concrete class, the temperatures during the 39 min heating are listed in Table 5, in which *T*_NCC-1_, *T*_NCC-2_, *T*_RCC-1_, and *T*_RCC-2_ represent the measured temperature of specimens NCC-1, NCC-2, RCC-1, and RCC-2, respectively.

**Table 5:** The temperature and corresponding ratio at measure points in the 39-min heating.

Measure Point	*T*_NCC-1_ (°C)	*T*_NCC-2_ (°C)	*T*_NCC-2_/*T*_NCC-1_	*T*_RCC-1_ (°C)	*T*_RCC-2_ (°C)	*T*_RCC-2_/*T*_RCC-1_
Point 1	590.0	560.2	0.949	482.5	503.6	1.044
Point 2	44.2	52.4	1.186	23.1	27.6	1.194
Point 3	13.2	18.6	1.409	14.7	16.6	1.129

The analysis in Figure 7 and Figure 8 and Table 5 shows that:

(1) Before the 39 min of heating, the curves of “measured temperature to time” of the four specimens appear very similar. The temperature variation at the cross section for both the normal RC columns and the RAC columns shows no significant difference.
(2) The heating rate is slower and the fire resistance duration is longer for RAC column RCC-1 compared to normal concrete column NCC-2, which has almost the same concrete strength rating. This could be attributed to the greater porosity and water absorption in RCAs, causing greater moisture content, which may in turn consume more heat through water evaporation [24].
(3) The fire resistance performance of lower concrete strength columns with the same cross section is better than that of higher concrete strength columns under the same initial axial force ratio. Because of the lower permeability with increased strength of concrete, higher concrete strength is more susceptible to spalling and results in lower fire resistance [25].
(4) The variation tendency of heating curves from Measure Point 1, which is very close to the outside of the columns, is similar to that from ISO834 [21] in Figure 3, while the curves from Measure Point 2, which is further from the outside of the columns, are significantly different from that of ISO834 [21]. As seen in Table 5, the temperatures at Point 3 were not affected by the fire at such a short heating time of 39 min because Point 3, which is in the center of the column section, is far from the fire.
(5) Due to the rapid proliferation of internal cracks inside the columns through heating, at around 40 min heating, the heating rate at Measure Point 2 for normal concrete specimen NCC-1 and NCC-2 increases, while at Point 2 around 120 min and at Point 3 around 100 min for specimen NCC-1 the increase accelerates.
(6) As shown in Table 5, there are six ratio values of *T*_NCC-2_/*T*_NCC-1_ and *T*_RCC-2_/*T*_RCC-1_ in total for four specimens, with five of them greater than 1. The temperature for higher concrete strength columns at the same measure points and same moment is slightly higher, but the difference is small.

#### 3.1.3. Vertical Axial Displacement

**Figure 9:**
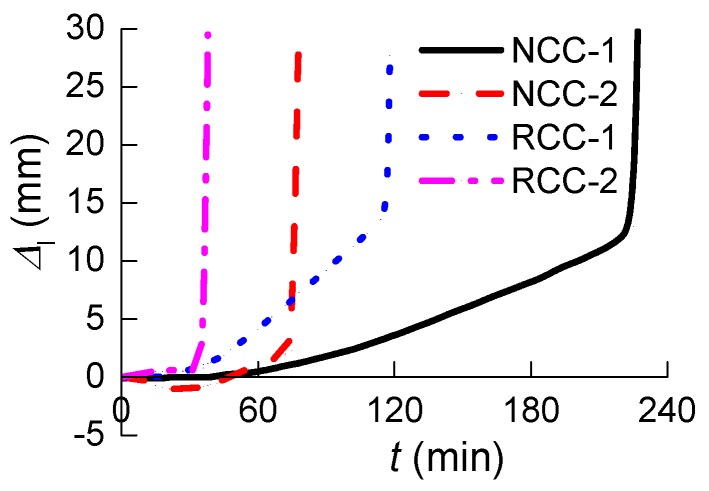
Specimen “vertical displacement to time” curves.

Figure 9 shows the curves of “vertical axial displacement to time” of the four specimens under the coupling effects of constant axial force and high temperature, in which ∆*_l_* is the axial displacement of columns, positive values indicate columns in a state of axial compression, and negative values indicate a state of extension. It can be seen from Figure 9 that:

(1) The normal RC column NCC-2 shows axial extension before 48 min, while the columns NCC-1, RCC-1, and RCC-2 do not. The expansion of inner air voids in the vertical axial direction under the pressure of trapped expanding steam during heating made the axial extension for column NCC-2. Similar axial extension happened for columns under heating test in Miao’s paper [26]. The extension for NCC-2 reaches its maximum value of 1.1 mm at 27 min, corresponding to a strain of 0.00367, which is almost the ultimate strain of concrete. After the internal air was released from the microcrack, the value of axial extension for NCC-2 returns to 0 at about 48 min. The column started to shorten and cracks developed.
(2) The RAC columns RCC-1 and RCC-2 exhibited no axial extension. The fire resistance performance of RCC-1 is better than that of RCC-2: Specimen RCC-2 deformed sharply and was seriously damaged at 39 min heating, while this happened 79 min later (at 118 min) for RCC-1. The cracks on the column with higher concrete strength started earlier because of its greater axial compression force *N* under the similar axial force ratio *n*; *N* can be seen in Table 3. Then the cracks led to a greater rate of temperature transmission to the interior of the columns, which resulted in a gradual deterioration of the inner concrete. During the test, the axial compression force remained unchanged, which means the axial force ratio *N* in Equation (1) remained constant. The actual area of the cross section of columns reduced during the test because the concrete started to crack and some material was shed under heating, which means *f*_c_ and *A*_c_ in Equation (1) became smaller and the actual axial force ratio *n* became greater. This is the key factor in the sharp deformation of columns—the damage which occurred under the combined effect of heating and axial load.
(3) The concrete strength of the normal RC column NCC-2 is very close to that of the RAC column RCC-1. The axial displacement was only 2.3 mm at 78 min for NCC-2, then the axial deformation increased sharply and the column exhibited sudden damage. The same occurred to RCC-1 at 118 min, with an axial displacement of 15 mm. The duration of effective fire resistance for specimen RCC-1 is therefore 51.28% longer than that of NCC-2. The greater porosity in the RCAs enables the column RCC-1 to exhibit greater deformation under heating. This clearly shows that the RAC column has better fire resistance performance than the normal RC column.

#### 3.1.4. Lateral Deflection

**Figure 10:**
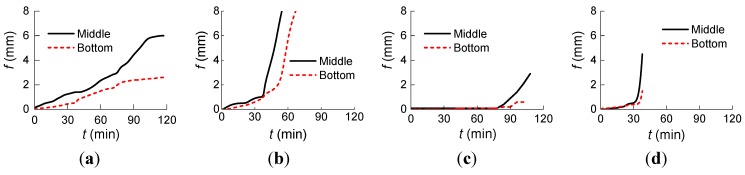
Curves of “lateral deflection to time” of specimens: (**a**) NCC-1; (**b**) NCC-2; (**c**) RCC-1; and (**d**) RCC-2.

In order to measure the lateral deflection of the columns in horizontal direction, two asbestos-wrapped molybdenum wires were attached at the bottom and the middle of the columns, as shown in Figure 2b. Figure 10 shows the relationship of “lateral deflection to time” of the specimens.

Table 6 shows the lateral deflection and their ratios of specimens at different times. In Figure 10 and Table 6, *f* is the lateral deflection, *f*_1_ and *f*_2_ are the lateral deflection at the middle and the bottom, respectively. The lateral deflection at the middle height of column NCC-2 reached 198.5 mm during failure.

**Table 6:** Lateral deflection and their ratios of specimens at different times.

Specimen	NCC-1			NCC-2			RCC-1			RCC-2		
Time-point (min)	40	80	120	25	50	60	40	80	118	15	27	39
*f*_1_ (mm)	1.40	3.00	6.00	0.50	4.00	8.00	0.12	0.20	2.90	0.13	0.40	4.50
*f*_2_ (mm)	1.00	1.70	2.60	0.30	1.50	2.20	0.10	0.15	0.60	0.10	0.25	1.50
*f*_1_/*f*_2_	1.40	1.76	2.31	1.67	2.67	3.64	1.20	1.33	4.83	1.30	1.60	3.00

Figure 10 and Table 6 show that:

(1) The developing rate of lateral deflection of all the specimens became faster with the increase of heating time.
(2) The ratio of *f*_1_ to *f*_2_ increased more quickly at the later period with the increase of heating time.
(3) Compared to the normal RC column NCC-2 with a similar concrete strength, the lateral deflection of RAC column RCC-1 was smaller at the earlier heating and the deformation developing was relatively stable because more heat needed to be consumed by the greater moisture content in recycled aggregates; this induced less severe deformation in the specimen.
(4) The concrete on the top part of column RCC-1 cracked and the column failed while there were no lateral displacements during the first 90 min of the fire at the bottom and middle part of the column.

#### 3.1.5. Failure Characteristics

**Figure 11:**
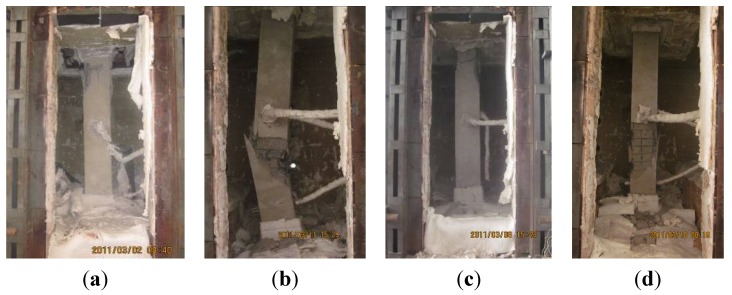
Global failure modes of specimens (**a**) NCC-1; (**b**) NCC-2; (**c**) RCC-1; and (**d**) RCC-2.

Figure 11 shows the global failure modes of the specimens after testing.

**Figure 12:**
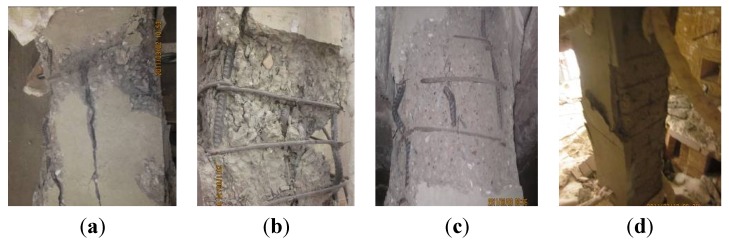
Local failure modes of specimens: (**a**) upper 1/3 of NCC-1; (**b**) lower part of NCC-2; (**c**) upper 1/3 of RCC-1; and (**d**) lower part of RCC-2.

Figure 12 shows the severely damaged local failure modes of the specimens.

Cracks developing at the lower part of the columns on fire could be observed through an observation port on the furnace. We observed the concrete spalling on NCC-2 and RCC-2 because it occurred just at the area we could see. Unfortunately, we could not see spalling on columns NCC-1 and RCC-1 because it happened at the top area of the columns, as shown in Figure 11, which was not within the observation port area.

**Figure 13:**
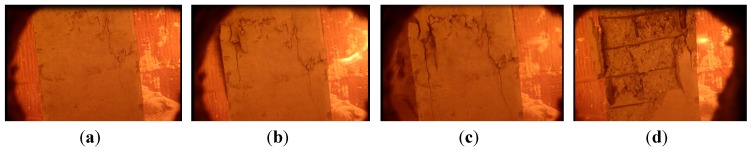
Concrete spalling observed from the observation port at different moments for RCC-2: (**a**) 36 min 40 s; (**b**) 36 min 47 s; (**c**) 36 min 51 s; and (**d**) 36 min 54 s.

Due to the non-homogeneity of concrete, which gives each specimen a unique material structure and a unique distribution of voids and moisture inside the concrete mass, the damage process of each specimen under heating is different and can be described as follows:

(1) Specimen NCC-1. The observed cracks occurred at about 39 min. It could be observed through an observation port on the furnace that the dense cracks appeared at about 60 min. The bared coarse aggregate turned whiter and then the column failed at 227 min. There was a vertical crack with a maximum width of 2 mm running through the height of the column on the four sides. The concrete dropped from a large area, about 300 mm to 130 mm along the height from the top. The steel bars buckled to 50 mm at this position, which is 600 mm from the top.
(2) Specimen NCC-2. The observed cracks occurred at about 28 min. Dense cracks appeared at about 40 min. The lateral deflection started to accelerate at 60 min and the column failed at 78 min. The maximum lateral deflection reached 198.5 mm. The concrete dropped at the area of 200 mm to 1110 mm from the bottom. The steel bars were exposed and buckled to 60 mm.
(3) Specimen RCC-1. The observed cracks occurred at around 50 min. The cracks stretched visibly at about 90 min, and the specimen failed at 118 min. The concrete cover, which was 700 mm from the top, separated from the column and the inside steel bars were exposed. A vertical crack with a maximum width of 2 mm ran along the height of the column. The steel bars buckled to 50 mm, 350 mm down from the top of the column.
(4) Specimen RCC-2. The observed cracks occurred at about 30 min. The cracks stretched visibly at about 35 min. At 36 min 51 s, concrete spalling occurred on the column over a large area, 50 mm to 1100 mm up from the bottom, and the inside steel bars were exposed. Figure 13 shows the concrete spalling process at different moments. Then the specimen failed at 39 min. A vertical crack with a maximum width of 2 mm ran through the height of the column and all the steel bars buckled to 50 mm.

### 3.2. Finite Element Method Analysis on Temperature Field of Concrete Columns

#### 3.2.1. Thermal Performance Parameters

The thermal performance parameters of concrete include bulk density, ρ; thermal conductivity, λ_c_; and specific heat capacity, *c*_c_. The fire temperature changes with the heating time, and the thermal performance parameters of the material change with the fire temperature. The temperature field analysis of a structure is a nonlinear transient heat conduction problem.

For this paper, the cross section analysis of the temperature field was independently analyzed according to [15]. The parameters of the thermal performance were determined as follows:

Thermal conductivity λ_c_, W/(m·°C).

When the RCA replacement is 0%:

(2) $${\text{λ}}_{\text{c}}=1.556-0.24\left(T/120\right)+0.012{\left(T/120\right)}^{2}\;20{}^{\circ}\text{C}\leq T\leq 1200{}^{\circ}\text{C}$$

When the RCA replacement is 100%:

(3) $${\text{λ}}_{\text{c}}=1.38-0.24\left(T/120\right)+0.012{\left(T/120\right)}^{2}\;20{}^{\circ}\text{C}\leq T\leq 1200{}^{\circ}\text{C}$$

Specific heat capacity *c*_c_, J/(kg·°C).

When the RCA replacement is 0%:

(4) $$c_{\text{c}}=892.\text{5+80}\left(T/120\right)-4{\left(T/120\right)}^{2}\;20{}^{\circ}\text{C}\leq T\leq 1200{}^{\circ}\text{C}$$

When the RCA replacement is 100%:

(5) $$c_{\text{c}}=935\text{+80}\left(T/120\right)-4{\left(T/120\right)}^{2}\;20{}^{\circ}\text{C}\leq T\leq 1200{}^{\circ}\text{C}$$

Bulk density ρ, (kg/m^3^).

The concrete bulk density decreases slightly as temperature increases, but the change in value is insignificant. Thus, it can be a considered as a constant in the analysis of the temperature fields of RAC components. The value of concrete bulk density changes from 2200 kg/m^3^ to 2400 kg/m^3^. The bulk density for normal concrete is 2400 kg/m^3^ and for RAC is 2300 kg/m^3^ in this paper.

#### 3.2.2. Finite Element Method

**Figure 14:**
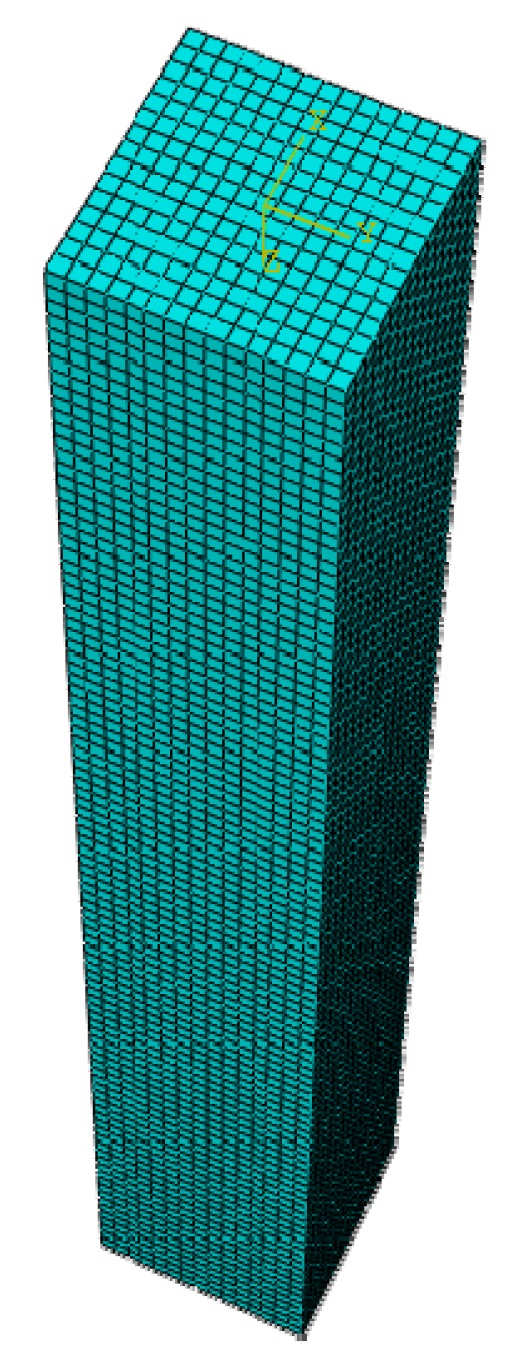
Mesh of the model.

Virtual components were designed with ABAQUS Software (ABAQUS Inc., Provindence, RI, USA), the thermal conductivity of the material was set in software, and the mass density and specific heat parameters were determined thereby. The measured furnace temperature was used as a heating parameter. The four lateral sides of columns were set as the fire surfaces, while the top and the bottom sides were set as the adiabatic surfaces. The eight-node continuous heat conduction 3D solid element DC3D8s were used and meshed as shown in Figure 14. Related studies have shown that the impact of reinforcements on the temperature field need not be considered during such analysis when the concrete reinforcement ratio is less than 4% [27]. The reinforcement ratio here is 1.24%, so we consider the temperature of steel bars to be the same as the encasing material.

#### 3.2.3. Finite Element Method Calculating and Analysis

**Figure 15:**
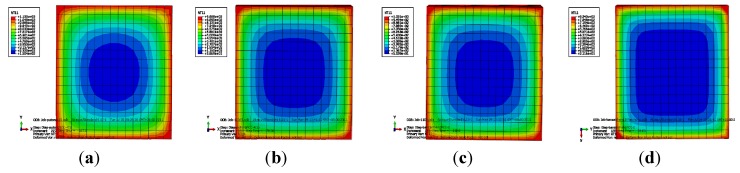
Moire pattern of temperature of specimens: (**a**) NCC-1; (**b**) NCC-2; (**c**) RCC-1; and (**d**) RCC-2.

Figure 15 shows the moire pattern of the cross section temperature for the four specimens.

Analysis from the calculated results shows that: (1) the temperature field at the four corners of the cross section exhibits arc-shaped distribution due to the bi-directional nature of the fire source; (2) a significant temperature gradient exists between the (hot) surface and (cooler) interior of the cross section; and (3) the area exhibiting higher temperatures spread inward gradually as the fire time increased.

**Figure 16:**
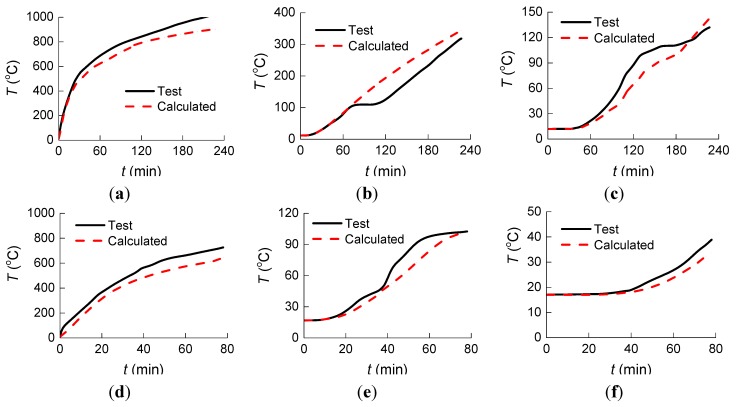

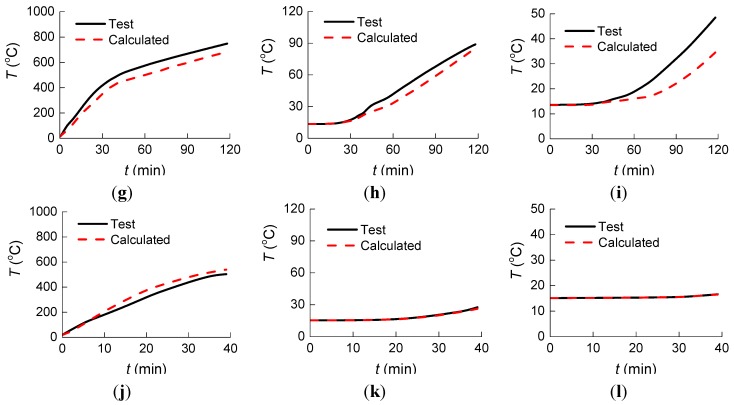
Comparison of temperature between calculated and measured results at the three measure points. NCC-1: (**a**) Measure Point 1; (**b**) Measure Point 2; and (**c**) Measure Point 3. NCC-2: (**d**) Measure Point 1; (**e**) Measure Point 2; and (**f**) Measure Point 3. RCC-1: (**g**) Measure Point 1; (**h**) Measure Point 2; and (**i**) Measure Point 3. RCC-2: (**j**) Measure Point 1; (**k**) Measure Point 2; and (**l**) Measure Point 3.

Figure 16 shows a comparison of temperatures between results from ABAQUS Software and measured results at the three measure points. They agree very well.

Table 7 shows the test and calculated temperatures, as well as the relative error at different measure points at 39 min. Analysis from Table 7 shows that the calculated temperature at each test point agrees well with the measured results.

**Table 7:** Test and calculated temperature, as well as relative error at different measure points at 39 min.

Specimen measure point	NCC-1			NCC-2			RCC-1			RCC-2		
	1	2	3	1	2	3	1	2	3	1	2	3
Test temperature (°C)	590.0	44.2	13.2	560.2	52.4	18.6	482.5	23.1	14.7	503.6	27.6	16.6
Calculated temperature (°C)	543.7	43.4	12.8	478.9	48.2	18.0	429.9	22.3	14.5	539.8	26.2	16.5
Relative error (%)	−7.8	−1.8	−3.0	−14.5	−8.0	−3.2	−10.9	−3.5	−1.4	7.2	−5.1	−0.6

## 4. Conclusions

(1) The rate of heat transfer from the surface to the inside of the column increases with compressive strength for both RAC columns and normal RC columns during fire conditions.
(2) Under the same initial axial force ratio, for both RAC columns and normal RC columns with the same cross section, those with lower concrete compressive strengths demonstrate better fire resistance performance. The fire resistance performance decreases as the concrete compressive strength increases, due to its higher density and lower porosity, which cause cracks to appear earlier and the material to degrade more rapidly.
(3) The fire resistance performance of RAC columns is better than that of the normal concrete columns with the same concrete compressive strength. This is because the rate of temperature penetration for RAC columns is lower than that of normal RC columns under nominal fire condition heating.
(4) Finite element method (FEM) analysis on the temperature fields of both the normal concrete columns and RAC columns, which showed almost the same results as the experiments, is able to be used to avoid the expensive test cost.
(5) The RAC columns may be applied in practical projects.
